# New Similarity of Triangular Fuzzy Number and Its Application

**DOI:** 10.1155/2014/215047

**Published:** 2014-03-20

**Authors:** Xixiang Zhang, Weimin Ma, Liping Chen

**Affiliations:** ^1^Business & Management School, Tongji University, Shanghai 200092, China; ^2^Jiaxing University, Jiaxing 314001, China

## Abstract

The similarity of triangular fuzzy numbers is an important metric for application of it. There exist several approaches to measure similarity of triangular fuzzy numbers. However, some of them are opt to be large. To make the similarity well distributed, a new method SIAM (Shape's Indifferent Area and Midpoint) to measure triangular fuzzy number is put forward, which takes the shape's indifferent area and midpoint of two triangular fuzzy numbers into consideration. Comparison with other similarity measurements shows the effectiveness of the proposed method. Then, it is applied to collaborative filtering recommendation to measure users' similarity. A collaborative filtering case is used to illustrate users' similarity based on cloud model and triangular fuzzy number; the result indicates that users' similarity based on triangular fuzzy number can obtain better discrimination. Finally, a simulated collaborative filtering recommendation system is developed which uses cloud model and triangular fuzzy number to express users' comprehensive evaluation on items, and result shows that the accuracy of collaborative filtering recommendation based on triangular fuzzy number is higher.

## 1. Introduction

Due to the uncertainty of information and the complexity of the decision-making problem, it is difficult for decision-makers to express their preferences by using exact numbers. It is easier for them to use linguistic labels (fuzzy terms) to express their preferences [1, 2]. Triangular fuzzy number not only can be used to express the vagueness and the uncertainty of information, but also can be used to represent fuzzy terms in information processing. Besides integrated with decision-making [3–5], triangular fuzzy number has been applied in many fields such as risk evaluation [6], performance evaluation [7], forecast [8], matrix games [9], and space representation [10].

Similarity is an important tool to provide the foundation for analogical reasoning between two fuzzy concepts, and is widely applied in many fields [9]. Some applications of triangular fuzzy numbers depend on their similarity. Chen and Lin proposed the distance of two triangular fuzzy numbers and calculated the similarity of two triangular fuzzy numbers based on the distance between them [11]. Hsieh and Chen put forward the concept of graded mean integration representation and computed the similarity of two triangular fuzzy numbers based on it [12]. Yager used the kernel function to represent a triangular fuzzy number [13], and Xu defined the concept of fuzzy expectation value of a fuzzy number [14]. By using the utility value of triangular fuzzy number, Hsieh and Chen compared two triangular fuzzy numbers and measured the similarity of two triangular fuzzy numbers [15]. S.-J. Chen and S.-M. Chen (2003) presented a simple center of gravity method to calculate the center-of-gravity (COG) point of a triangular fuzzy number and put forward an approach to express the similarity of two triangular fuzzy numbers based on COG [16]. Wan proposed a new method to express the similarity of two triangular fuzzy numbers and applied it to multisensor data fusion field to recognize multisensor objectives [17]. Sadi-Nezhad et al. used vector similarity to measure the similarity of triangular fuzzy numbers [10].

The shape of a triangular fuzzy number is an important metric; however, it was not taken into consideration before. Therefore, a new method SIAM (Shape's Indifferent Area and Midpoint) to measure the similarity of two triangular fuzzy numbers is proposed, which considers the shapes and midpoints of them. To compare the effect of different methods, experiment is carried out to calculate the similarity of triangular fuzzy numbers, and result shows that similarity based on the proposed method is better distributed. Then, the triangular fuzzy number is used to express the user's comprehensive evaluation of items in collaborative filter recommendation. Case comparison and simulation are carried out; results indicate that collaborative filter recommendation based on triangular fuzzy number can obtain better accuracy.

## 2. Related Works

### 2.1. Triangular Fuzzy Number and Its Operators

If a regular convex fuzzy set $\tilde{M}$ is based on the real domain and meets the following conditions: (1) there exists only one element *x*_0_ that $\mu_{\tilde{M}}(x_{0})=1$, (2) $\mu_{\tilde{M}}(x)$ is continuous, then $\tilde{M}$ is a fuzzy number, which means “a real number that is approximated to *x*_0_.” $\tilde{M}$ can be expressed as follows [15]:

(1) $$\begin{array}{c} \mu_{\tilde{M}}\left(x\right)=\{\begin{array}{cc} L\left(x\right), & l\leq x\leq m,\\ R\left(x\right), & m\leq x\leq r. \end{array} \end{array}$$

*L*(*x*) is an increasing function and continuous on the right, *R*(*x*) is a decreasing function and continuous on the left, and 0 ≤ *L*(*x*), *R*(*x*) ≤ 1. When *L*(*x*) is expressed as (*x* − *l*)/(*m* − *l*) and *R*(*x*) is represented as (*x* − *r*)/(*m* − *r*), $\mu_{\tilde{M}}(x)$ is called a triangular fuzzy number (TFN). To donate it simply, the left threshold value *a* ^*l*^, the midpoint *a* ^*m*^, and the right threshold value *a* ^*u*^ are used to represent a triangular fuzzy number $\tilde{A}$, $\tilde{A}=(a^{l},a^{m},a^{u})$, and its membership function is as follows:

(2) $$\begin{array}{c} \mu_{\tilde{M}}\left(x\right)=\{\begin{array}{cc} \frac{x-a^{l}}{a^{m}-a^{l}}, & a^{l}\leq x\leq a^{m},\\ \frac{x-a^{u}}{a^{m}-a^{u}}, & a^{m}\leq x\leq a^{u},\\ 0, & \text{otherwise}. \end{array} \end{array}$$

Let $\tilde{A}=(a^{l},a^{m},a^{u})$ and $\tilde{B}=(b^{l},b^{m},b^{u})$ be two triangular fuzzy numbers; the basic operators for triangular fuzzy numbers are as follows:

(3) A~⊕B~=(al,am,au)⊕(bl,bm,bi)=(al+bl,am+bm,au+bu),A~⊗B~=(al,am,au)⊕(bl,bm,bi)=(albl,ambm,aubu),1A~=(1au,1am,1al),mA~=m(al,am,au)=(mal,mam,mau).

### 2.2. Similarity of Triangular Fuzzy Numbers

A similarity measure is an important tool for presenting a degree of similarity between two objects. The application of triangular fuzzy number in different area needs different measurements of its similarity.

Chen and Lin proposed a method to calculate similarity based on distance of midpoint and boundary. Let $\tilde{A}=(a^{l},a^{m},a^{u})$ and $\tilde{B}=(b^{l},b^{m},b^{u})$ be two triangular fuzzy numbers, the similarity of them can be denoted as follows:

(4) $$\begin{array}{c} S_{\text{MB}}\left(\tilde{A},\tilde{B}\right)=1-\frac{\left|a^{l}-b^{l}\right|+\left|a^{m}-b^{m}\right|+\left|a^{u}-b^{u}\right|}{3}. \end{array}$$

Hsieh and Chen presented the concept of utility value of a triangular fuzzy number and used it to measure the similarity of triangular fuzzy numbers [15].

Suppose that there are *n* triangular fuzzy numbers, and all of them match the membership function ${\tilde{A}}_{i}=(a_{i}^{l},a_{i}^{m},a_{i}^{u})$, *i* = 1,2,…, *n*. Then the maximizing set *T* and minimizing set *B* with membership function are as follows:

(5) $$\begin{array}{c} \mu_{T}\left(x\right)=\{\begin{array}{cc} \frac{x-x_{\min}}{x_{\max}-x_{\min}}, & x_{\min}\leq x\leq x_{\max}\\ 0, & \text{otherwise}, \end{array}\\ \mu_{B}\left(x\right)=\{\begin{array}{cc} \frac{x-x_{\max}}{x_{\min}-x_{\max}}, & x_{\min}\leq x\leq x_{\max}\\ 0, & \text{otherwise}. \end{array} \end{array}$$

Let $\tilde{A}=(a^{l},a^{m},a^{u})$ be a triangular fuzzy number. Then its right utility value $\mu_{T}({\tilde{A}}_{i})$ based on maximizing set *T* and its left utility value $\mu_{B}({\tilde{A}}_{i})$ based on minimizing set *G* are

(6) μT(A~i)=sup⁡(μA~i(x)∧μT(x))=(aiu−xmin⁡)(xmax⁡−xmin⁡)−(aim−aiu),μB(A~i)=sup⁡(μA~i(x)∧μG(x))=(xmax⁡−ail)(xmax⁡−xmin⁡)−(ail−aim).

And the total utility value is $\mu_{\text{UV}}({\tilde{A}}_{i})$:

(7) $$\begin{array}{c} \mu_{\text{UV}}\left({\tilde{A}}_{i}\right)=\frac{\mu_{T}\left({\tilde{A}}_{i}\right)+1-\mu_{B}\left({\tilde{A}}_{i}\right)}{2}. \end{array}$$

Suppose $\tilde{A}=(a^{l},a^{m},a^{u})$ and $\tilde{B}=(b^{l},b^{m},b^{u})$ are two triangular fuzzy numbers. The utility similarity $S_{\text{UV}}(\tilde{A},\tilde{B})$ between them is as follows:

(8) $$\begin{array}{c} S_{\text{UV}}\left(\tilde{A},\tilde{B}\right)=\frac{U_{\text{UV}}\left(\tilde{A}\right)\times U_{\text{UV}}\left(\tilde{B}\right)}{\max\left({\left(U_{\text{UV}}\left(\tilde{A}\right)\right)}^{2},{\left(U_{\text{UV}}\left(\tilde{B}\right)\right)}^{2}\right)}, \end{array}$$

where $U_{\text{UV}}(\tilde{A})$ and $U_{\text{UV}}(\tilde{B})$ are the utility values of $\tilde{A}$ and $\tilde{B}$, respectively. And $S_{\text{UV}}(\tilde{A},\tilde{B})=1$ when $U_{\text{UV}}(\tilde{A})$ and $U_{\text{UV}}(\tilde{B})$ are both 0.

Based on the idea of graded mean integration-representation distance (GMIR), Hsieh and Chen (1999) presented a similarity measure for two triangular fuzzy numbers. The measurement of GMIR is as follows [12]:

(9) $$\begin{array}{c} S_{\text{GMIR}}\left(\tilde{A},\tilde{B}\right)=\frac{1}{1+d\left(\tilde{A},\tilde{B}\right)}, \end{array}$$

where

(10) $$\begin{array}{c} d\left(\tilde{A},\tilde{B}\right)=\left|P\left(\tilde{A}\right)-P\left(\tilde{B}\right)\right|,\\ P\left(\tilde{A}\right)=\frac{a^{l}+4a^{m}+a^{u}}{6},\;\;P\left(\tilde{B}\right)=\frac{b^{l}+4b^{m}+b^{u}}{6}. \end{array}$$

Yager presented the concept of kernel of fuzzy number [13]. Let $\left(\tilde{A},\mu_{\tilde{A}}(x)\right)$ be a fuzzy number and its kernel function can be denoted as follows:

(11) $$\begin{array}{c} K\left(\tilde{A}\right)=\frac{\int_{0}^{1}x\mu_{\tilde{A}}\left(x\right)dx}{\int_{0}^{1}\mu_{\tilde{A}}\left(x\right)dx}. \end{array}$$

Hou and Wu used the above expression to calculate the kernel function of triangular fuzzy number and obtained the same result as graded mean integration-representation [18]. Meanwhile, Xu put forward the method to calculate the expectation value of a triangular fuzzy number and also got the same result [14].

S.-J. Chen and S.-M. Chen presented a simple center method to calculate the center-of-gravity (COG) points of generalized fuzzy numbers, and put forward a similarity measure of triangular fuzzy numbers based on the center-of-gravity [16]. Let $\tilde{A}=(a^{L},a^{M},a^{U})$ be a triangular number and $\left(x_{\tilde{A}}^{*},y_{\tilde{A}}^{*}\right)$ the center-of-gravity; its formula is shown as follows:

(12) $$\begin{array}{c} x^{*}=\frac{x_{1}+x_{2}+x_{3}}{3},\;\;y^{*}=\frac{y_{1}+y_{2}+y_{3}}{3}. \end{array}$$

Let $\tilde{A}=(a^{l},a^{m},a^{u})$ and $\tilde{B}=(b^{l},b^{m},b^{u})$ be two triangular fuzzy numbers, and let $\left(x_{\tilde{A}}^{*},y_{\tilde{A}}^{*}\right)$ and $\left(x_{\tilde{B}}^{*},y_{\tilde{B}}^{*}\right)$ be their COGs; the similarity of $\tilde{A}$ and $\tilde{B}$ can be defined as follows:

(13) SCOG(A~,B~)=(1−|al−bl|+|am−bm|+|au−bu|3) ×(1−|xA~∗−xB~∗|)B(A~,B~)∗min⁡⁡(yA~∗,yB~∗)max⁡⁡(yA~∗,yB~∗),

where $x_{\tilde{A}}^{*}$, $y_{\tilde{A}}^{*}$, $x_{\tilde{B}}^{*}$, and $y_{\tilde{B}}^{*}$ are calculated according to formula (13) and $B(\tilde{A},\tilde{B})$ is used to determine if the COG distance is considered; the new formula is defined as follows:

(14) $$\begin{array}{c} B\left(\tilde{A},\tilde{B}\right)=\{\begin{array}{cc} 1, & \text{if}\;S_{\tilde{A}}+S_{\tilde{B}}>0\\ 0, & \text{if}\;S_{\tilde{A}}+S_{\tilde{B}}=0, \end{array} \end{array}$$

where $S_{\tilde{A}}$ and $S_{\tilde{B}}$ are the lengths of the triangular fuzzy number $\tilde{A}$ and $\tilde{B}$, respectively, defined as follows:

(15) $$\begin{array}{c} S_{\tilde{A}}=a^{u}-a^{l},\\ S_{\tilde{B}}=b^{u}-b^{l}. \end{array}$$

It has been proven that the similarity based on COG has the following property: $S_{\text{COG}}(\tilde{A},\tilde{B})=1$ if and only if $\tilde{A}=\tilde{B}$.

Because of the complexity of objective things and the disturbance of the measurement surroundings, there exist uncertainty and fuzziness in dealing with multisensor data fusion (MSDF). Wan (2011) used triangular fuzzy number to express its uncertainty and fuzziness [17]. What is more, multiattribute decision-making theory was used to solve problems in MSDF and a new multisensor object recognition method was proposed. Thus, another similarity of two triangular fuzzy numbers is presented. Suppose $\tilde{A}=(a^{l},a^{m},a^{u})$ and $\tilde{B}=(b^{l},b^{m},b^{u})$ are two triangular fuzzy numbers; their similarity is defined as follows:

(16) $$\begin{array}{c} S_{\max}\left(\tilde{A},\tilde{B}\right)=\frac{\sum_{i\in \{l,m,u\}}a^{i}b^{i}}{\max\left(\sum_{i\in \{l,m,u\}}a^{i}a^{i},\sum_{i\in \{l,m,u\}}b^{i}b^{i}\right)}. \end{array}$$

To solve multiple criteria group decision-making (MCGDM) problems, method based on vector similarity is used to measure similarity of two triangular fuzzy numbers [6]:

(17) $$\begin{array}{c} S_{\text{VS}}\left(\tilde{A},\tilde{B}\right)=\frac{2\sum_{i\in \{l,m,u\}}a^{i}b^{i}}{\sum_{i\in \{l,m,u\}}a^{i}a^{i}+\sum_{i\in \{l,m,u\}}b^{i}b^{i}}. \end{array}$$

## 3. Similarity of Triangular Fuzzy Numbers Based on SIAM

Triangular fuzzy numbers are used to represent uncertain and incomplete information in decision-making, risk evaluation, and expert systems. Measurement of similarity should keep some parameters of triangular fuzzy numbers. Shape and midpoint are important metrics of a triangular fuzzy number. Therefore, they should be taken into consideration when measuring the similarity of triangular fuzzy numbers.

Let a triangular fuzzy number move horizontally and overlap its midpoint with the midpoint of another triangular fuzzy number. The overlapped part of two triangular fuzzy numbers is indifferent, and the ratio of the overlap part can be used to represent the shape similarity of two triangular fuzzy numbers. And the distance between the midpoints of two triangular fuzzy numbers can express the difference of them.

**Figure 1:**
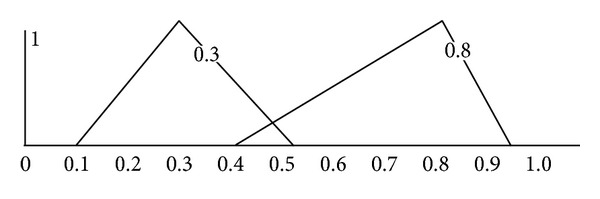
Two triangular fuzzy numbers $\tilde{A}=(0.1,0.3,0.5)$ and $\tilde{B}=(0.4,0.8,0.9)$.

Suppose $\tilde{A}=(0.1,0.3,0.5)$ and $\tilde{B}=(0.4,0.8,0.9)$ are two triangular fuzzy numbers.  Figure 1 shows these two triangular fuzzy numbers. These two triangular fuzzy numbers have different shapes and midpoints.

**Figure 2:**
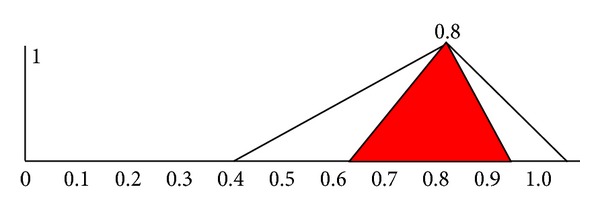
The intersection area of ${\tilde{A}}^{\prime }=(0.6,0.8,1.05)$ and $\tilde{B}=\left(0.4,0.8,0.9\right)$.

Move $\tilde{A}=(0.1,0.3,0.5)$ to ${\tilde{A}}^{\prime }=(0.6,0.8,1.0)$ so that the midpoint of ${\tilde{A}}^{\prime }$ is identical with the midpoint of $\tilde{B}$. The intersection area of ${\tilde{A}}^{\prime }$ and $\tilde{B}$ can be demonstrated in the red area in Figure 2. The intersection area of ${\tilde{A}}^{\prime }$ and $\tilde{B}$ is indifferent, so the intersection area is called the indifferent area.

The ratio of the indifferent area can be used to express the shape similarity of two triangular fuzzy numbers. Thus, a new method to measure the similarity of two triangular fuzzy numbers based on shape and the midpoint is obtained.

Suppose $\tilde{A}=(a^{l},a^{m},a^{u})$ and $\tilde{B}=(b^{l},b^{m},b^{u})$ are two triangular fuzzy numbers; move $\tilde{A}=(a^{l},a^{m},a^{u})$ to ${\tilde{A}}^{\prime }=(a^{l}+(b^{m}-a^{m}),b^{m},a^{u}+(b^{m}-a^{m}))$, so that the midpoint of ${\tilde{A}}^{\prime }$ is identical with $\tilde{B}$. Since $\tilde{A}$ and ${\tilde{A}}^{\prime }$ have the same shape, and ${\tilde{A}}^{\prime }$ is used to represent $\tilde{A}$, the ratio of their shapes' indifferent area of $\tilde{A}$ and $\tilde{B}$ can be defined as follows:

(18) SSIA(A~,B~)=2RA~′∩B~RA~+RB~=2(min⁡⁡(bu,au+(bm−am))−max⁡⁡(al+(bm−am),bl))(au−al)+(bu−bl),

where *a* ^*u*^ − *a* ^*l*^ ≠ 0 and *b* ^*u*^ − *b* ^*l*^ ≠ 0; the similarity of triangular fuzzy numbers based on their shape's indifferent area and midpoint can be described as follows:

(19) SSIAM(A~,B~)=α(1−|am−bm|)+βSSIA(A~,B~),α+β=1, α>0,  β>0.

Similarity SIAM has the following properties:

1. reflexivity, that is, $S_{\text{SIAM}}(\tilde{A},\tilde{A})=1$;
2. symmetry, that is, $S_{\text{SIAM}}(\tilde{A},\tilde{B})=S_{\text{SIAM}}(\tilde{B},\tilde{A})$.

**Theorem 1**

Let $\tilde{A}=(a^{l},a^{m},a^{u})$ and $\tilde{B}=(b^{l},b^{m},b^{u})$ be two triangular fuzzy numbers and $S_{\text{ SIAM }}(\tilde{A},\tilde{B})=S_{\text{ SIAM }}(\tilde{B},\tilde{A})=1$ if and only if $\tilde{A}=\tilde{B}$.

**Proof**

If $\tilde{A}=\tilde{B}$, then *a* ^*l*^ = *b* ^*l*^, *a* ^*m*^ = *b* ^*m*^, and *a* ^*u*^ = *b* ^*u*^. $S_{\text{SIAM}}(\tilde{A},\tilde{B})=1$ is true. If $S_{\text{SIAM}}(\tilde{A},\tilde{B})=1$, then 1 − |*a* ^*m*^ − *b* ^*m*^| = 1 and $S_{\text{SIA}}\left(\tilde{A},\tilde{B}\right)=1$. As 1 − |*a* ^*m*^ − *b* ^*m*^| = 1 means *a* ^*m*^ = *b* ^*m*^, $S_{\text{SIAM}}(\tilde{A},\tilde{B})=2\left(\min(b^{u},a^{u})-\max(a^{l},b^{l})\right)/\left(\left(a^{u}-a^{l})+(b^{u}-b^{l}\right)\right)=1$ is obtained. According to Figure 2, we can see that *S*_SIAM_ = 1 only and if only *a* ^*l*^ = *b* ^*l*^, *a* ^*u*^ = *b* ^*u*^. Thus, $\tilde{A}=\tilde{B}$ is true. Therefore, we can conclude that $S_{\text{SIAM}}(\tilde{A},\tilde{B})=1$ if and only if $\tilde{A}=\tilde{B}$.

There are several approaches to measure the similarity of two triangular fuzzy numbers. *S*_MB_ measures the similarity based on the distance of left point, midpoint, and right point. *S*_UV_ calculates the similarity based on the utility value. *S*_GMIR_ gets the similarity based on graded mean integration-representation distance. *S*_GOV_ obtains the similarity based on the center-of-gravity of triangular fuzzy numbers. *S*_max⁡_ uses vector cosine to compute the similarity. And *S*_SIAM_ utilizes the shape's indifferent area and midpoint to measure the similarity of two triangular fuzzy numbers. From the equations, we conclude that *S*_MB_, *S*_GMIR_, *S*_max⁡_, and *S*_SIAM_ are easier to calculate while *S*_GOV_ and *S*_UV_ are more difficult.

**Figure 3:**
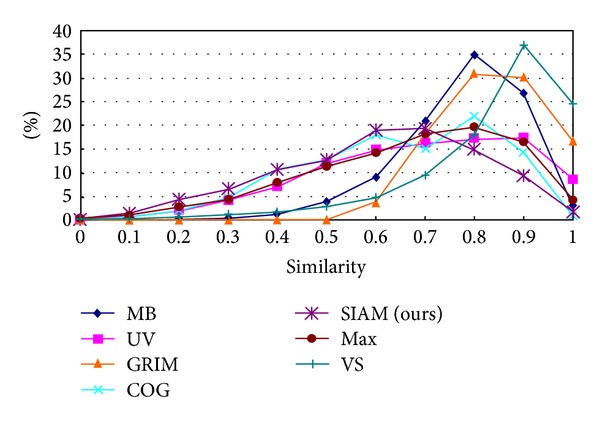
Similarity distribution of the different approaches.

To compare the distribution of different approaches that measure similarity of triangular fuzzy numbers, a simulation program is written which generates randomly 10000 pairs of triangular fuzzy numbers and measures the similarity of them based on different approaches. To compare the similarity distribution of each approach, the similarity is dealt with as follows: multiply the calculated similarity by 10, adds 0.5, and omits decimal fractions and it turns into an integer between 0 and 10; (2) counts the numbers of the integers, respectively, and divides the integers by 10; and (3) computes the ratio of each similarity interval.  Figure 3 shows the result of the ratio of each similarity interval based on different method to measure similarity of triangular fuzzy numbers.

From Figure 3, we can see that *S*_VS_, *S*_MB_, and *S*_GMIR_ are oriented to larger. *S*_max⁡_, *S*_SIAM_, *S*_GOV_, and *S*_UV_ can obtain distributed result more normally. *S*_SIAM_ is much easier according to the complexity of the measuring equation, and it gets better normally distributed result.  Table 1 shows the detail of the similarity distribution rate of different approaches.

## 4. Its Application in Collaborative Filtering Recommendation

Collaborative filtering technique has been widely applied [19–21], which recommends items to a user based on the rating information of its neighbors.

The basic principle of collaborative filtering recommendation assumes that users will have similar evaluation on other items if they have similar evaluation on some items. Thus, items can be recommended to a user based on its nearest neighbours' evaluation. Therefore, similarity measurement based on users' evaluation on items is the key problem in collaborative filtering recommendation systems.

Comprehensive measurement, such as evaluation ranking frequency vector and cloud model (CM), can be used to represent users' comprehensive evaluation and to calculate users' similarity [22].

Cloud model is a cognitive model proposed by Liu et al. which can synthetically describe the randomness and fuzziness of concepts and implement the uncertain transformation between a qualitative concept and its quantitative instantiations [23]. Zhang et al. analysed the effect of evaluation ranking frequency vector in collaborative filtering recommendation system and used cloud model to express the users' comprehensive evaluation on items; simulation on MoviesLens dataset was carried out to prove that collaborative filtering recommendation based on cloud model obtained better performance than that of ranking frequency vector [22]. Therefore, the paper compares the performance of collaborative filtering recommendation based on cloud model and triangular fuzzy number, respectively.

If a user's evaluation on traded items is *X* = {*x*_1_, *x*_2_,…, *x*_*n*_}, the user's comprehensive evaluation on items can be represented by cloud model *V* = (*Ex*, *En*, *He*):

(20) $$\begin{array}{c} Ex=\overset{-}{X}=\frac{1}{n}\sum_{i=1}^{n}x_{i},\\ He=\sqrt{\frac{\pi }{2}}\times \frac{1}{n}\sum_{i=1}^{n}\left|x_{i}-E_{x}\right|,\\ En=\sqrt{S^{2}-\frac{1}{3}H_{e}^{2}}. \end{array}$$

The triangular fuzzy number shows the fuzzy and uncertainty of information, so it is appropriate to represent the user's comprehensive evaluation on items. The average of the user's comprehensive evaluation on items represents the midpoint of a triangular fuzzy number, namely, the degree of concentration on items. The absolute value of user's evaluation on item minus the average of user's evaluation on items is called the fuzzy discretization (FD) of user's evaluation, that is, the uncertainty of user's evaluation. Therefore, if a user evaluates the items in *X* = {*x*_1_, *x*_2_,…, *x*_*n*_}, its comprehensive evaluation can be expressed by a triangular fuzzy number $\tilde{A}=(a^{L},a^{M},a^{U})$.

(21) $$\begin{array}{c} a^{M}=\frac{1}{5n}\sum_{i=1}^{n}x_{i},\\ a^{L}=a^{M}-\frac{1}{2}\left(\text{FD}-E\right),\\ a^{U}=a^{M}+\frac{1}{2}\left(\text{FD}+E\right),\\ \text{FD}=\frac{1}{5n}\sum_{i=1}^{n}\left|x_{i}-a^{M}\right|,\\ E=\frac{1}{5n}\sum_{i=1}^{n}\text{sig}\left(x_{i}-a^{M}\right). \end{array}$$

### 4.1. Comparison of Users' Comprehensive Evaluation

Take the example from Zhang et al. [22]. Four users, A, B, C, and D, evaluated 10 items, respectively.  Table 2 shows the detailed evaluation of each user on the items.

Bellogín et al. [20] found that the similarity based on evaluation ranking frequency vector was in [0.98, 1] and it is difficult to distinguish its nearest neighbours. They put forward the cloud model to express the user's evaluation on items. The similarity of users based on the cloud model is as shown in Table 3.

There does not exist much difference between the users' similarity based on cloud model. We use (21) to express users' comprehensive evaluation on items; the four users' comprehensive evaluation on items can be represented by the following triangular fuzzy numbers, respectively: $\tilde{A}=(0.2128,0.28,0.3088)$, $\tilde{B}=(0.8,0.9,1)$, $\tilde{C}=(0.7504,0.84,0.878)$, and $\tilde{D}=(0.291,0.32,0.387)$. According to (19), the similarity of four users is obtained; Table 4 shows the result.

From the case, we can see that the similarity based on triangular fuzzy number has better discrimination. Users A and B have different evaluation on items; the similarity based on cloud model is 0.956, which is not consistent with our intuitionistic judging. While the similarity based on triangular fuzzy numbers is 0.461, which means that there is difference between the evaluation of users A and B. Therefore, it can be concluded that it is reasonable to use a triangular fuzzy number to express users' evaluation on items and calculate their similarity based on it.

### 4.2. Simulation in Collaborative Filtering Recommendation System

The simulated system is developed by using Visual Studio.NET. The experiment takes data from MoviesLens (http://www.grouplens.org/), which is a recommendation system for research and provides recommendation list to users based on users' evaluation on movies. The experiment uses the MoviesLens 100 K data set, which contains 100000 records of 943 users' evaluation on 1648 items. 80 percent of the data set is used as history data and 20 percent of it is chosen as test data set.

Accuracy is a metric to evaluate the recommendation quality. And mean absolute error (MAE) can be used to represent recommendation accuracy, which calculates the deviation between the user's forecasted score and the user's real score. The smaller the MAE, the better the performance of a recommendation system.

Assume the users' forecasted score set of a recommendation system is *F* = {*f*_1_, *f*_2_,…, *f*_*n*_}, and the users' real score set is *R* = {*r*_1_, *r*_2_,…, *r*_*n*_}. The MEA can be donated as the following formula:

(22) $$\begin{array}{c} \text{MAE}=\frac{1}{n}\sum_{i=1}^{n}\left|f_{i}-r_{i}\right|. \end{array}$$

**Figure 4:**
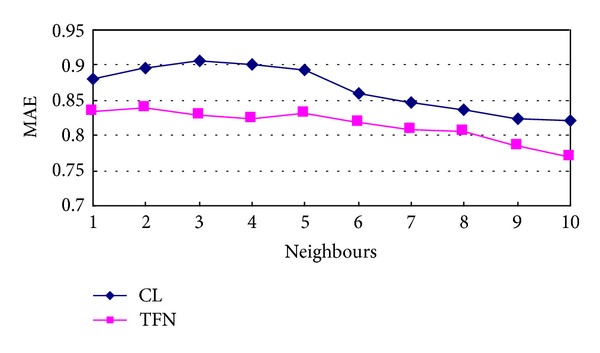
MAE based on CL and TFN.

A simulated collaborative filtering recommendation system is developed. Cloud model and triangular fuzzy number are used to express the comprehensive users' evaluation on items in the simulated system, similarity of users is calculated based on them, and items are recommended to users based on the nearest *k* neighbours' scores on items. To score the performance of collaborative filtering recommendation, the MAE is used to measure recommendation accuracy. Simulation result is shown in Figure 4.

Figure 4 shows that the more neighbours are used in collaborative filtering recommendation, the more accuracy the recommendation system can obtain. From the simulation, the MAE of collaborative filtering recommendation based on triangular fuzzy number can be more accurate than that on cloud model.

## 5. Conclusion

Triangular fuzzy number has been applied in many fields such as risk analysis, decision-making, and evaluation. A new method to measure the similarity of two triangular fuzzy numbers is proposed, which measures the similarity based on the shape's indifferent area and midpoints of two triangular fuzzy numbers. Comparison with other measurements shows that the similarity measuring method proposed in this paper can be distributed more normally.

Finally, triangular fuzzy number is applied in collaborative filtering recommendation system, in which triangular fuzzy number is used to express the users' comprehensive evaluation on items. Case demonstration and simulation on MoviesLens show that triangular fuzzy number can express users' comprehensive evaluation on items and the collaborative filtering recommendation accuracy can be higher.

## Figures and Tables

**Table 1:** Similarity distribution rate of different approaches.

Similarity interval	MB	UV	GRIM	COG	SIAM (ours)	max	VS
[0,0.05)	0	0.2	0	0	0.13	0.24	0.2
[0.05,0.15)	0	0.74	0	0.62	1.43	1.07	0.16
[0.15,0.25)	0.03	1.99	0	1.91	4.3	2.71	0.63
[0.25,0.35)	0.33	4.15	0	4.73	6.54	4.35	1.09
[0.35,0.45)	1.09	7.18	0	10.52	10.62	7.92	1.62
[0.45,0.55)	3.84	11.9	0.01	12.5	12.72	11.34	2.79
[0.55,0.65)	9.03	14.76	3.65	17.9	18.93	14.16	4.73
[0.65,0.75)	20.83	16.11	18.6	15.05	19.31	18.12	9.42
[0.75,0.85)	35.05	17.15	30.89	21.97	14.91	19.57	17.92
[0.85,0.95)	26.7	17.32	30.22	14.22	9.35	16.47	36.89
[0.95,1]	3.1	8.5	16.63	0.58	1.76	4.05	24.55

**Table 2:** Users' evaluation on 10 items.

User	I1	I2	I3	I4	I5	I6	I7	I8	I9	I10
A	2	1	1	1	2	1	1	2	1	2
B	5	4	5	4	5	4	5	4	5	4
C	4	5	3	4	5	5	4	4	5	3
D	2	1	2	2	1	1	2	2	1	2

**Table 3:** Users' similarity based on CM.

Similarity	A	B	C	D
A	1	0.956	0.965	**0.999**
B	0.956	1	**0.999**	0.967
C	0.965	**0.999**	1	0.975
D	**0.999**	0.967	0.975	1

**Table 4:** Users' similarity based on TFN.

Similarity	A	B	C	D
A	1	0.461	0.522	**0.728**
B	0.461	1	**0.892**	0.516
C	0.522	**0.892**	1	0.489
D	**0.728**	0.516	0.489	1
